# Sequence Inversion to Facilitate Concurrent Radiotherapy and Systemic Therapy. A Proof of Principle Study in the Setting of a Phase II Randomized Trial in Prostate Cancer

**DOI:** 10.3389/fonc.2020.570660

**Published:** 2020-09-30

**Authors:** Derek Wilke, Lori Wood, Slawa Cwajna, Robert Rutledge, Helmut Hollenhorst, David Bowes, Nikhilesh Patil, Casely T. Ago, Jean-Philippe Pignol

**Affiliations:** ^1^Department of Radiation Oncology, Dalhousie University, Halifax, NS, Canada; ^2^Division of Medical Oncology, Department of Internal Medicine, Dalhousie University, Halifax, NS, Canada

**Keywords:** radiation, docetaxel (Compound CID: 148124), prostate cancer, randomized trial, hormone therapy

## Abstract

**Background:** Concomitant chemo-radiation for pelvic cancers remains challenging to be delivered at full doses. We hypothesized that fewer delays in chemotherapy would occur if the sequence of radiotherapy would be reversed, starting with the boost volume followed by the elective nodal volume. We report the result of a Phase II randomized study for high risk prostate cancer.

**Patients and Method:** The study was a double-blinded phase II randomized trial. Patients were eligible if they had non-metastatic high-risk prostate cancer. All patients received 2.5 years of hormonal therapy and 46.5 Gy in 25 fractions to the pelvic lymph nodes. Patients received a radiation boost to the prostate, either before or after whole pelvic irradiation. Concurrent (20 mg/m^2^) Docetaxel was given on the first day of radiotherapy and weekly thereafter for a total of eight treatments until predefined toxicity stopping rules.

**Results:** Ninety patients were included and randomized. Four were ineligible for the analysis. In total, 42 patients were randomized to the standard sequence, 44 patients to the experimental sequence. There were statistically fewer GI or GU toxicities leading to a docetaxel dose reduction or omission in the experimental sequence compared to the standard sequence, 5 vs. 15 events (*p* = 0.027). There was no difference in overall survival, cause-specific survival, or biochemical-relapse free survival between the two sequences.

**Conclusions:** This is the first study to test sequence inversion for pelvic radio-chemotherapy in a randomized double-blind trial. Less chemotherapy interruptions or dose reductions occurred by inverting the radiation sequence of the large field and the boost.

The trial was registered with Clinicaltrials.gov: NCT00452556

## Background

Chemo-radiation is a standard treatment for several pelvic malignancies, including those arising from the cervix, rectum, and anal canal, and has also been tested for high risk prostate cancer (1–6). When regional nodes are included in the radiotherapy field, and systemic therapy itself has bowel or urinary toxicity, often there are delays or dose-reductions, in one or both modalities, and either treatment can be interrupted or even discontinued. However, by tradition, the radiotherapy treatment consists of sequentially large fields treated at low doses, followed with a boost on the gross target volume. It is unknown if the order of treatment between boost and large field may have an impact on treatment tolerance and could enable fewer delays or dose-reductions of chemotherapy, by postponing the time when patients will present with significant bowel side effects.

In developed countries, prostate cancer is the most frequent non-cutaneous cancer in men (7). Twenty percent are classified as high risk, meaning they have a 30–50% risk of nodal involvement (8) and a 20–30% chance of microscopic distant metastases (9). For those high-risk patients, treatment options include radiotherapy, usually combined with hormonal therapy, and less frequently surgery in some highly selected patients. Chemotherapy and concomitant chemo-radiation, which could target both the loco-regional disease and the distant micro-metastasis remains experimental. Among various systemic therapies used in prostate cancer, Docetaxel is a radio-sensitizer with activity against prostate cancer (10–12). However, Docetaxel is also known to cause gastrointestinal toxicity (13), so there is concern that when delivered concomitantly to radiotherapy this would lead to excessive, dose-limiting toxicity. In a study on 22 patients, Kumar et al. (14) showed that when delivered concomitantly, the full chemotherapy regimen could only be given in 50% of patients largely because of an excess of gastro-intestinal toxicities.

We hypothesized that there would be fewer dose reductions or delays in docetaxel chemotherapy if the sequence of radiotherapy would be reversed, starting with the boost volume followed by the large elective nodal volume. This is a proof of principle study of sequence inversion, using prostate cancer as an example. In this manuscript, we report the result of a phase II randomized study of concomitant chemoradiation in high risk prostate cancer patients comparing the standard sequence treating the large volume followed by the boost volume, with an experimental sequence delivering the boost dose before the loco-regional treatment. Outcomes include the number of dose reductions and/or delays in docetaxel, as a result of gastrointestinal or genitourinary toxicity, survival, and health-related quality of life measured by the Expanded Prostate Cancer Index Composite (EPIC) (15).

## Materials and Methods

### Eligible and Ineligible Patients

Patients were eligible if they had a high risk prostate cancer, defined in the current trial either as untreated patients with a 2003 TNM clinical stage T2c, T3a, or T3b, or a Gleason score 8 to 10, or a PSA ≥ 20 μg/L but less than 50 μg/L (16), a life expectancy of at least 5 years, and an ECOG (Eastern cooperative oncology group) performance status of 0 or 1. Also, patients who had radical prostatectomy (RP) were offered to receive regional radiation as part of the study if they had more than a 50% chance of biochemical recurrence following the Kattan et al. (17) Nomogram. Post radical prostatectomy patients had to have a post-operative PSA of <1.0 μg/L and be able to start the study protocol within 6 months from surgery. In all cases, patients must have had no evidence of metastatic disease after screening bone scan, chest X-ray, and CT scan of the abdomen and pelvis, and adequate end organ function in terms of bone marrow, liver, and kidneys. Patients were excluded if they had a PSA > 50 μg/L, prior pelvic radiotherapy, grade ≥ 2 peripheral neuropathy, prior malignancy, or known hepatitis B or C.

### Study Design

The study was a single center double-blinded phase II randomized design. Patients were approached for the study during the initial consultation by the study co-ordinator, and after informed consent was obtained, they were referred for medical oncology consultation. When deemed eligible for combined chemo-radiation treatment, patients were randomized in a 1:1 ratio to treatment sequence at the time of registration into the study. Randomization was performed by a computer algorithm, using SAS software version 9.4 (Cary, NC), using the permuted block design, using block sizes of four and six patients. To ensure blinded assessment of toxicities, the attending radiation oncologist completed the delineation of the target volumes and organs at risk and approved the final plans for each phase. All subsequent quality assurance, including verification dosimetry and daily image guidance, was reviewed by an independent radiation oncologist involved in the study, but not in the patient's treatment nor the assessment of toxicity. The attending physician was responsible for assessing and scoring toxicity at the time of weekly review within the hospital and was blinded to patient treatment sequence. Prior to study initiation, approval was obtained from the institutional ethics review board, and the trial was registered with ClinicalTrials.gov.

### Treatment Protocol: Chemotherapy and Radiation Prescriptions

Patients underwent radiation simulation following institutional guidelines. In brief, patients were immobilized supine in a Vak-Loc (CIVCO, Coralville, Iowa) and CT simulation. A planning MRI was performed in the treatment position using a 1.5 T magnet without an endorectal coil. All patients, including those having had radical prostatectomy, had insertion of gold fiducial markers in the prostate or prostate bed. The clinical target volume (CTV) for the larger volume which included the pelvic nodes included the external and internal iliac vessels plus 7 mm except where vessels were in direct abutment to bone or muscle, where the CTV included only the vessel. The CTV for the larger volume included the nodes and either the prostate plus 3 mm, except at the prostate-rectal interface, or the prostate bed, from the bottom of the anastomosis, superiorly, to the inferior aspect of the proximal vas deferens. The CTV for the smaller boost volume included only the prostate bed, or the prostate plus any extraprostatic extension, if present. The planning target volume (PTV) was equal to the CTV plus 7 mm. The dose fractionation was intended to be biologically equivalent to 70 Gy in 35 fractions, with the elective nodal dose equivalent to 46 Gy in 23 fractions. All patients received 46.5 Gy in 25 fractions to the pelvic lymph nodes. Patients who had not had previous radical prostatectomy received a boost, either before, or after whole pelvic irradiation, to the prostate and to the seminal vesicles for T3b tumors to a dose of 26.78 Gy in 13 fractions. The boost dose was reduced to 20.6 Gy in 10 fractions, if patients had previous radical prostatectomy. There were no specified dose constraints to organs at risk. Plans were approved if 95% of the dose covered 98% of the PTV volume. All patients were treated with static port intensity-modulated radiotherapy (IMRT), using either five or seven ports, with six MV photons. All radiation plans were verified by Medical Physics prior to treatment as per institutional policy. Daily image guidance was performed with on-board kV imaging, matching to the fiducial markers.

All patients received hormonal therapy with leuprolide acetate 45 mg subcutaneously every 6 months. This was given 4 months prior to starting concurrent chemoradiation and continued for two years post-treatment. In addition, patients received 4 weeks of daily bicalutamide 50 mg at the time of the first leuprolide acetate administration. Concurrent Docetaxel was given on the first day of IMRT (week 16 of protocol therapy) and weekly thereafter for a total of eight treatments at a dose of 20 mg/m^2^ over 30 min. Chemotherapy was withheld for grade 3 or greater: diarrhea, thrombocytopenia, absolute neutrophil count (ANC) < 500× 10^9^/L, febrile neutropenia with ANC < 1.0 × 10^9^/L, nausea, and vomiting, stomatitis, grade 2 peripheral neuropathy, or abnormal liver function tests. When acceptable toxicity was reached, docetaxel was restarted at 16 mg/m^2^. If grade 3 toxicity recurred, or if docetaxel was delayed more than 2 weeks, docetaxel was discontinued.

### Outcomes

The primary endpoint of the study was the comparison between sequences of the patients' proportion experiencing Docetaxel dose reductions or omissions due to gastrointestinal or genitourinary toxicity. The number of dose reductions and/or delays was compared using Poisson regression. Secondary endpoints included the time to selected grade 2 and 3 NCI CTCAE version 3.0 toxicity, the difference in incidence of grade 2 and 3 toxicity, as well as the difference in overall bowel domain score of the EPIC at weeks 16, 20, and 24. Differences in rates of toxicity were compared using Poisson regression, when the modeling fit the data, and when the assumptions of Poisson regression were not met, the proportions of toxicity between sequences were calculated using the Chi-squared test. Differences in Overall survival and time to selected grades of toxicity were calculated using the Log Rank test, and differences in biochemical relapse-free survival and prostate cancer related mortality were calculated using competing risk proportional hazards modeling. The date of biochemical failure in the patients with no prior surgery occurred at the time the PSA reached a value of the PSA nadir +2, and the date of biochemical failure in the patients who had had a prior radical prostatectomy occurred at a PSA of 0.2. Statistical analyses were conducted using SAS software version 9.4 (Cary, NC).

### Sample Size

Based on the Kumar et al. (14) study, we assumed 50% of patients receiving the standard sequence would require docetaxel dose reductions or delays and 20% of patients in the experimental sequence would require dose reductions or delays. With a type 1 error rate of 0.05 and a power of 80%, 39 patients per sequence would be required. Assuming a 10% rate of withdrawal or discontinuation, 43 patients per sequence were calculated. Early trial stopping rules dictated that if there was a greater than 60% grade 2 or more gastrointestinal or genitourinary toxicity in the first 10 patients in either sequence, the dose of docetaxel would be reduced to 16 mg/m^2^ weekly in all subsequently treated patients, and if more than 30% of patients subsequently experienced grade 3 toxicity in the next 10 patients treated after the dose reduction, the study would have been discontinued.

## Results

### Patients Characteristics

Table 1 demonstrates that the two treatment sequences were well-balanced for baseline characteristics. In total 90 patients were registered and randomized. Four were deemed ineligible for the analysis, including three who withdrew consent, and one found to have an invasive bladder cancer on the planning MRI. In total, 42 patients were randomized to the standard sequence, 44 patients to the experimental sequence. The trial began recruitment June 7, 2007, and completed Jan 23, 2012, after the prespecified sample size requirements were met.

**Table 1:** Baseline characteristics.

**Characteristic**	**Standard sequence**	**Experimental sequence**	***p*-value**
*N*	42	44	
**Age**			
Median (years)	67.4	65.6	0.3
Range (years)	47–78	53–75	
**T category, Clinical [*****N*** **(%)]**			
T1c–T2a	8 (19)	11 (25)	0.83
T2b–T2c	8 (19)	5 (11)	
T3a–T3b	26 (62)	28 (64)	
**Gleason score, biopsy [*****N*** **(%)]**			
* ≤* 6	5 (12)	7 (16)	0.67
7	17 (40)	14 (32)	
8–10	20 (48)	23 (52)	
Previous radical prostatectomy [*N* (%)]	11 (26)	14 (32)	0.56
**Baseline PSA (mcg/L)**			
Median	11.25	10.28	0.67
Range	0.22–48.67	0.93–50	
**ECOG performance status [*****N*** **(%)]**			
0	36 (86)	40 (91)	0.51
1	6 (14)	4 (9)	

*N, number; T, Tumor; PSA, Prostate specific antigen; ECOG, Eastern cooperative oncology group*.

### Chemo-Radiation Delivery and Toxicity

All patients received radiotherapy, as per protocol, without breaks or dose reductions and the vast majority were able to receive the full dose of chemotherapy, There were significantly fewer GI or GU toxicities leading to a docetaxel dose reduction or omission in the experimental sequence compared to the standard sequence, 5 vs. 15 events (*p* = 0.027), using Poisson regression. Secondary study endpoints included the total amount of Docetaxel that can be delivered. In the standard sequence 78.6% of patients received 8 weeks of chemotherapy compared to 81.8% in the experimental sequence (*p* = 0.88), and 76.2% of patients did not require a docetaxel dose reduction compared to 77.2% in the experimental sequence (*p* = 0.88). Goodness of fit testing indicated that the Poisson regression fit the data well (*p* = 0.44) for the primary endpoint of the study.

Secondary endpoints also included individual GI and GU toxicities, selected ones of which are listed in Table 2. There were significantly more cumulative GI grade 2 and 3 toxicities in the experimental sequence, 91%, compared to the standard sequence, 69% (*p* = 0.0109). The rates of Grade 2 or higher diarrhea corresponding to 3 or more bowel movements per day above baseline were similar with the standard sequence, 50%, compared to 52.2% in the experimental sequence (*p* = 0.83). Conversely, there were more combined grade 2 and 3 gastrointestinal toxicities in the experimental sequence, with a non-statistically significant trend to more proctitis in the experiment sequence, 22.7 vs. 11.9% (*p* = 0.186). Constipation was the only statistically significant individual item (*p* = 0.045). There was a statistically significant delay in time to grade 1 diarrhea in the experimental sequence (*p* = 0.04). There was also a non-statistically significant trend to more combined grade 2 and 3 urinary frequency corresponding to 2 times increase of the normal voiding frequency, 70.5 vs. 50% (*p* = 0.0525).

**Table 2:** Selected GI and GU toxicities.

**Toxicity item**	NCI toxicity^*^	**Standard Sequence Arm**		**Experimental Sequence Arm**		**Individual item**	**Grouped items**
**Group**	**Grade 2 and 3**	**Number of events**	**Percent of patients**	**Number of events**	**Percent of patients**	***p*-value**	***p*-value**
Gastrointestinal	Incontinence, anal	0	0.00	6	4.55	0.005	Grade 2 and 3 CHISQ
	Proctitis	9	11.90	45	22.73	0.186	*p* = 0.011
	Diarrhea	60	50.00	78	52.27	0.833	Grade 3 CHISQ
	Constipation	0	0.00	2	4.55	0.045	*p* = 0.528
	Hemorrhage, GI	4	7.14	2	4.55	0.660	
Genito-urinary	Urinary frequency	139	50.00	205	70.45	0.053	Grade 2 and 3 CHISQ
	Cystitis	30	21.43	8	11.36	0.206	*p* = 0
	Incontinence, urinary	20	4.76	27	6.82	0.275	Grade 3 POISS
	Urinary retention	193	14.29	166	13.64	0.613	*p* = 0.056
	Hemorrhage, GU	10	4.76	11	4.55	0.617	

* *National Cancer Institute common toxicity criteria adverse event version 3.0, CHISQ, Chi-squared test; POISS, Poisson regression*.

It is important to note that bone marrow toxicity was infrequent in both sequences, with no patients experiencing febrile neutropenia (see Table 3). One patient in the experimental sequence had a lower GI bleed, requiring transfusion, but this resolved without further intervention. There were no treatment related deaths.

**Table 3:** Non-GI or GU toxicities.

**Toxicity item**	NCI toxicity^*^	**Standard Sequence Arm**		**Experimental Sequence Arm**		**Individual item**	**Grouped items**
**Group**	**Grade 2 and 3**	**Number of events**	**percent of patients**	**Number of events**	**percent of patients**	***p*-value**	***p*-value**
Sensori-motor	Neuropathy, sensory	0	0.00	2	2.27	0.102	
Bone marrow	Leukocytes	3	2.38	2	2.27	0.617	Grade 2 and 3 CHISQ
	Platelets	1	2.38	0	0.00	0.231	*p* = 0.4169
	Fever	1	2.38	0	0.00	0.231	
Liver	Alkaline phosphatase	0	0.00	4	2.27	0.021	
Allergy/Local reaction	Acute infusion reaction	14	7.14	4	4.55	0.617	

* *National Cancer Institute common toxicity criteria adverse event version 3.0, CHISQ = Chi-squared test*.

### Survival

**Figure 1:**
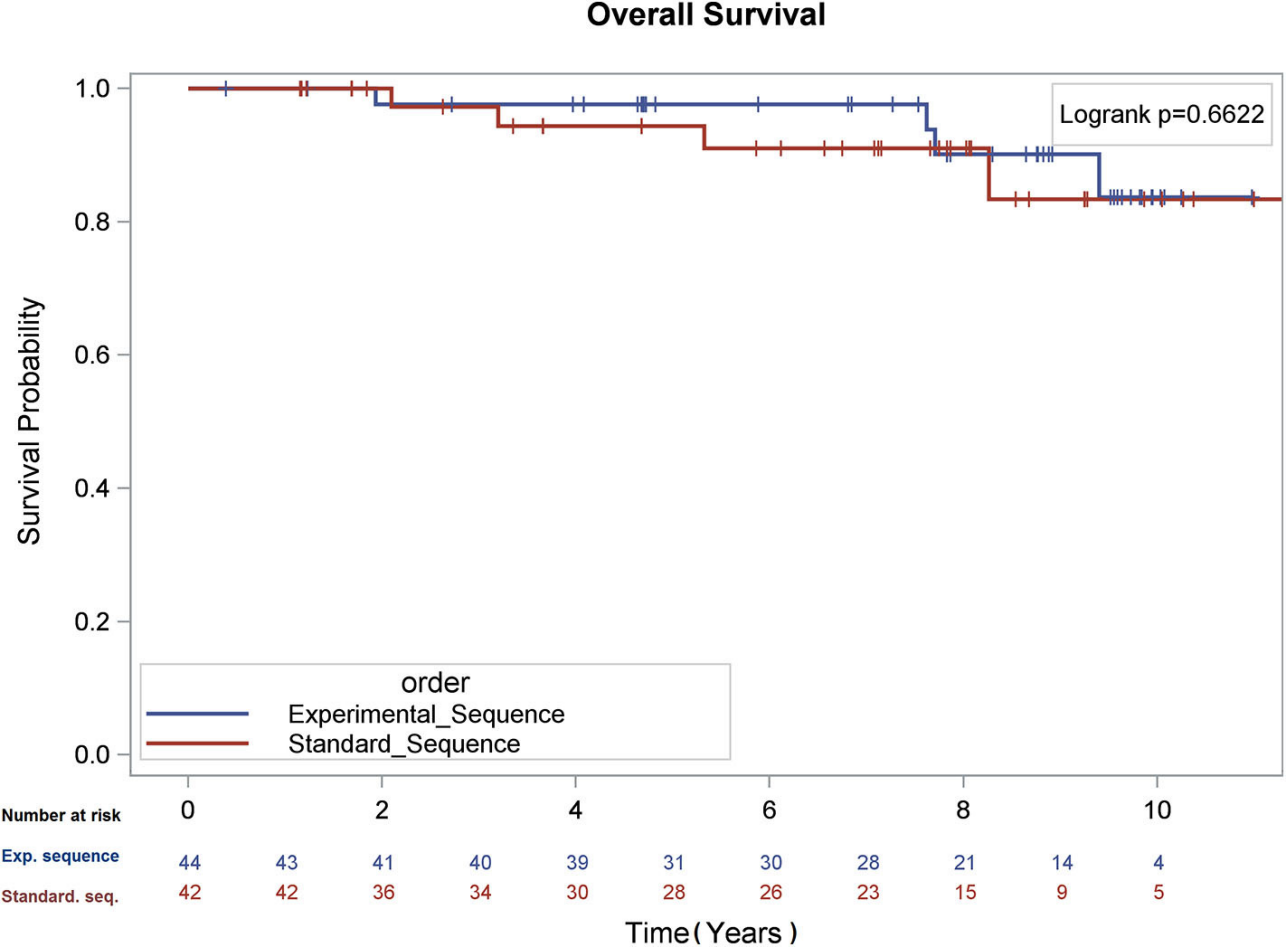
Kaplan – Meier analysis of overall survival.

**Figure 2:**
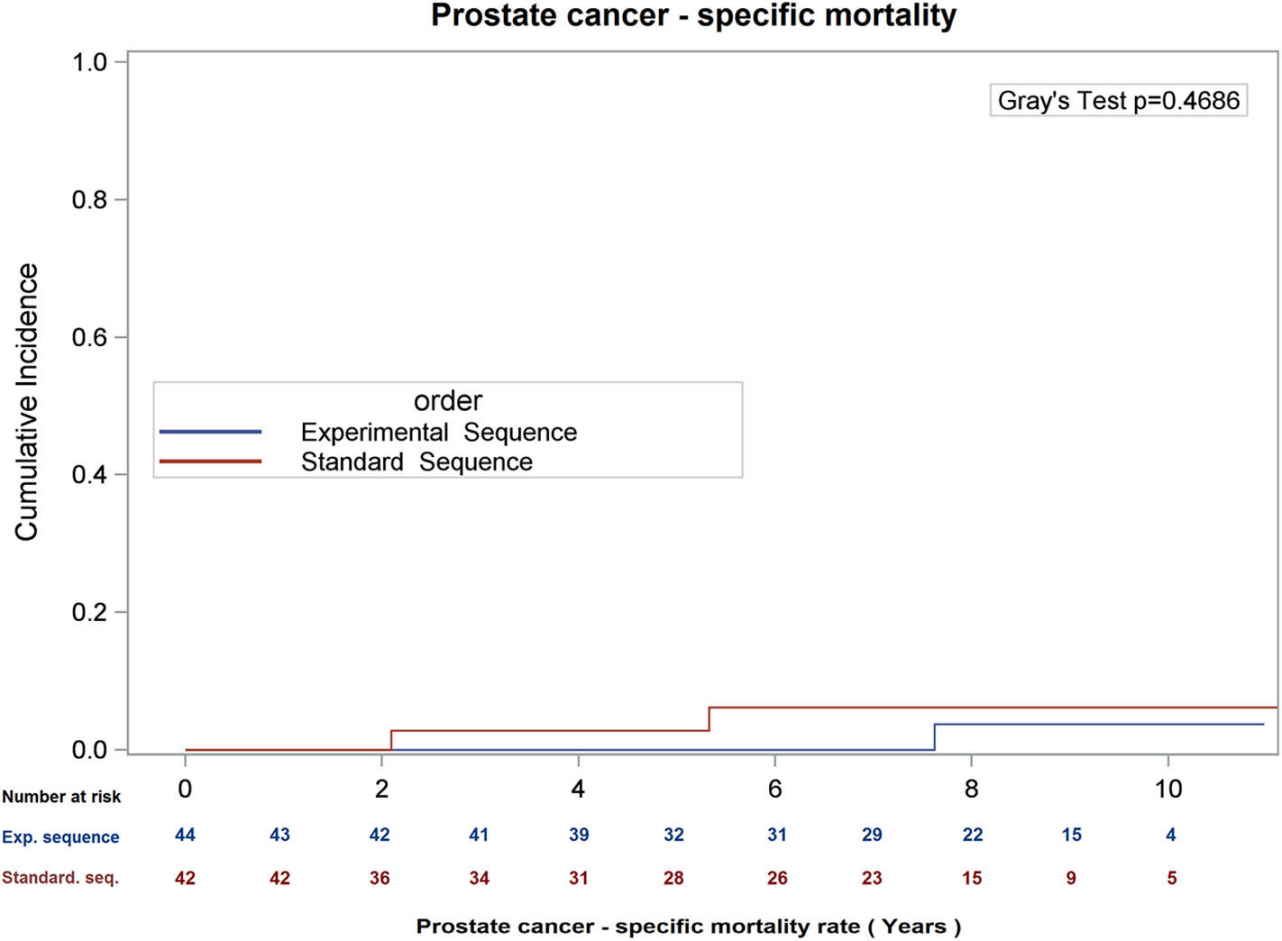
Cumulative incidence of prostate cancer – specific mortality, using competing risk analysis.

**Figure 3:**
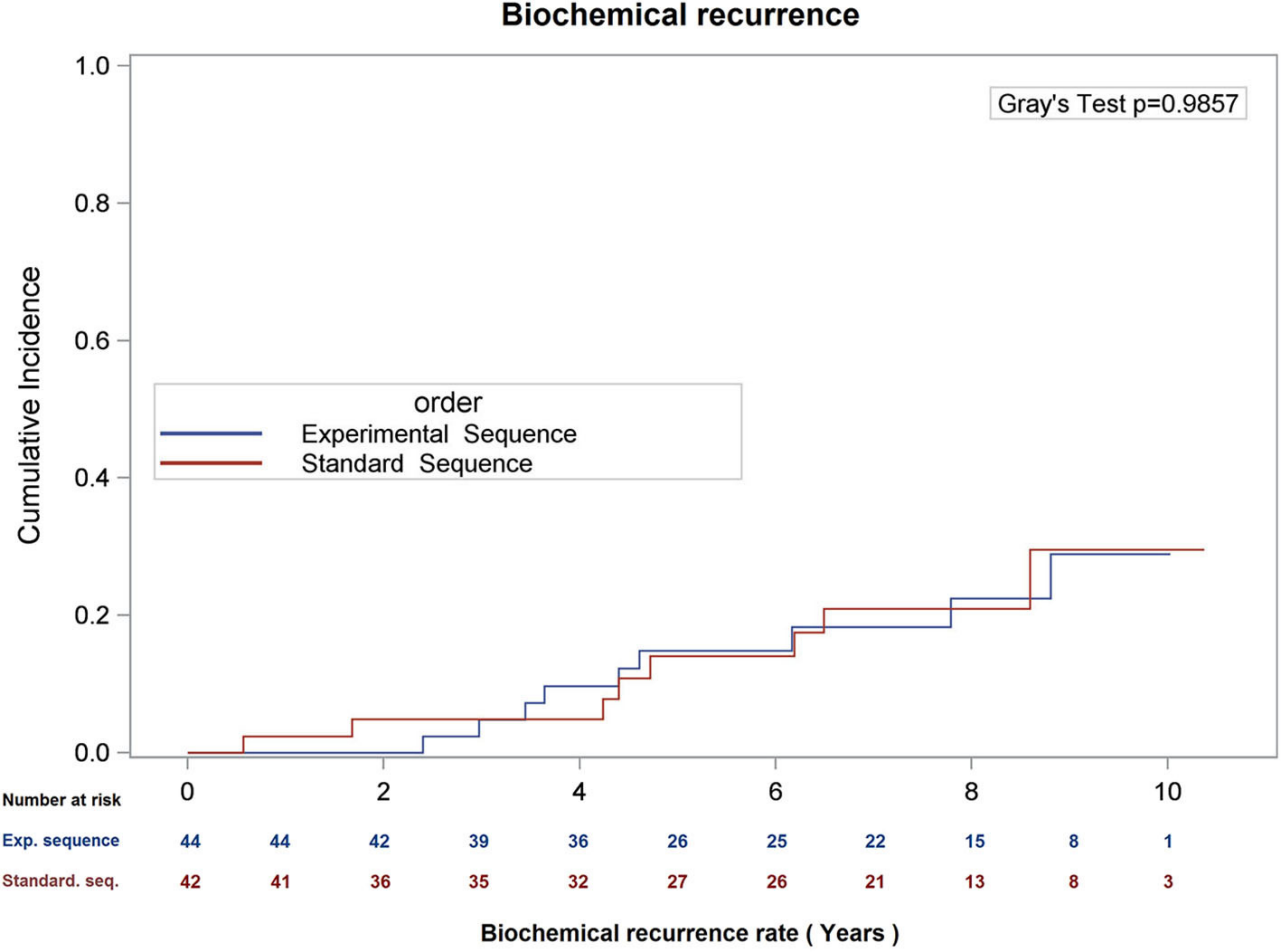
Cumulative incidence of biochemical recurrence (date of failure = PSA nadir + 2), using competing risk analysis.

There was no difference in overall survival (Log-Rank *p* = 0.66), cause-specific survival (Gray's Test *p* = 0.47), or biochemical recurrence (Gray's Test *p* = 0.98; see Figures 1–3) between the two sequences. However, overall survival is encouraging with 90% of patients alive at 8 years, and only 22.5 and 21.0% of patients demonstrating biochemical recurrence at 8 years, in the experimental sequence and standard sequences, respectively.

### Heath—Related Quality of Life

During the period of concurrent chemo-radiation (week 16–24 of protocol therapy) there was statistically significant declines in all domains of the EPIC, except on the Mental Component score of the SF12. For this domain there was a statistically decline in the standard sequence only using repeated measures analysis of variance (*p* = 0.01). For all the other domains, there was no difference between sequences in terms of score decline for the bowel, urinary, sexual, hormonal domains, nor in the AUA symptom scores, or Physical Component score.

## Discussion

This is the first proof of principle study, to test the inversion of the sequence of loco-regional radiation followed by the boost as the standard sequence or the reverse as the experimental sequence, in a randomized, double-blind trial. The study found that there were fewer dose-reductions or delays due to gastrointestinal or genitourinary toxicity when the radiotherapy started with the boost phase followed by the large loco-regional phase that includes the nodal volume. This could potentially enable more radio-sensitization of prostate cancer cells by docetaxel, and our study demonstrates that by inverting the order of the radiation sequences there was no detriment in cancer control to addressing only the grossly apparent tumor first. There was more grade 2 toxicity, mostly grade 2 urinary frequency, in the experimental sequence arm (70.4% vs. 50% combined grade 2 and 3 urinary frequency, *p* = 0.053, with 9.09% (four events in four patients) vs. 2.38% (one event in one patient) grade 3 urinary frequency, *p* = 0.18), but this excess was not sufficient to fit the pre-specified criteria to warrant chemotherapy dose reductions or delays, and resulted in 15 events resulting in dose reductions or delays in the standard sequence vs. five events in the experimental sequence, due to GU or GI toxicity. In essence, slightly more toxicity was seen in the experimental sequence, as a percentage of patient affected, but it didn't translate into more events that required reduction or omission of the systemic therapy, in the experiment arm. In fact, they had less events, and perhaps more bother, although this was not detected by bother as measured by the EPIC.

Clearly, however, docetaxel is out of favor to be combined with radiotherapy in a concurrent fashion, and is not recommended.

Similar sequence inversion of the boost and whole pelvic irradiation for concomitant chemo-radiation of prostate cancer with paclitaxel had been reported by Sanfilippo et al. (18), but this was not explored in a randomized study. Patients enrolled at the beginning of the study received the traditional sequence starting with whole pelvic lymphatics and prostate treated followed by a boost. Due to GI toxicity, the patients accrued later in the trial received inverted radiotherapy sequences starting with the prostate boost and treating the pelvic lymphatics last. The study reported a decrease in the incidence of grade 3 toxicity, however, a formal analysis of the toxicity rates before and after the sequencing switch was not provided. The patients in the Sanfilippo study also received biweekly paclitaxel and 9 months of androgen deprivation. They reported a 3 years biochemical-free relapse rate of 74%, using the Phoenix definition, and an 18% rate of grade 3 diarrhea, which is very similar to the 20.4% rate of grade 3 diarrhea in our study experimental sequence. Conversely, to Sanfilippo study we were able to escalate the loco-regional radiation dose to 46.5 Gy in 25 fractions with a similar rate of toxicity utilizing IMRT.

The strength in the present study is its randomized nature, blinding of participants and investigators, which limits potential bias. One other important aspect is that the incidence of grade 2 or higher bone marrow toxicity was less than 10%, without febrile neutropenia, likely owing to the weekly docetaxel regimen compared to docetaxel given every 3 weeks, when marrow suppression is more pronounced. Ideally, the optimal systemic agent would be radio-sensitizing only to tumor cells, would have a high degree of independent anti-tumor effect, and itself would not cause treatment-related diarrhea, or urinary toxicity.

What is the meaning for other cancer sites? The same sequence inversion strategy could be tested for anal canal, cervix, or rectal cancer or used as option when patients present with significant co-morbidities presenting a challenge to protocol completion.

In conclusion, our study demonstrates the proof of principle that sequence inversion of the large and boost volumes results in fewer dose deductions or delays in systemic therapy when there is overlapping normal tissue toxicity between the two. There was no detriment in cancer control to addressing only the grossly apparent tumor first. While the study will not turns the heels of radiotherapy on its head, it does provide scientific proof that in special circumstances it may offer an approach of how to optimize combined modality therapy, with radiotherapy and a systemic agent, when there is overlapping toxicity.

## Data Availability Statement

The raw data supporting the conclusions of this article will be made available by the authors, without undue reservation.

## Ethics Statement

The studies involving human participants were reviewed and approved by Nova Scotia Health Authority, Research Ethics Board. The patients/participants provided their written informed consent to participate in this study.

## Author Contributions

DW contributed to conceptualization, data curation, formal analysis, funding acquisition, investigation, methodology, project administration, resources, software, supervision, validation, visualization, writing—original draft, and writing—review and editing. LW contributed to conceptualization, data curation, funding acquisition, investigation, methodology, project administration, resources, supervision, validation, visualization, and writing—review and editing. SC contributed to data curation, investigation, project administration, and writing—review and editing. RR contributed to conceptualization, data curation, investigation, methodology, project administration, visualization, and writing—review and editing. HH contributed to conceptualization, data curation, investigation, methodology, project administration, and writing—review and editing. DB contributed to data curation, investigation, project administration, and writing—review and editing. NP contributed to data curation, investigation, project administration, and writing—review and editing. CA contributed to conceptualization, data curation, funding acquisition, investigation, methodology, project administration, resources, supervision, and writing—review and editing. J-PP contributed to formal analysis, investigation, methodology, project administration, resources, supervision, visualization, writing—original draft, and writing—review and editing. All authors contributed to the article and approved the submitted version.

## Conflict of Interest

DW reports grants and personal fees from Allergan, grants, and personal fees from AstraZeneca, grants, and personal fees from AbbVie, personal fees from Amgen, personal fees from Astellas, personal fees from Jansen, grants and personal fees from Paladin Labs, grants and personal fees from Sanofi, outside the submitted work. LW participates in advisory boards for BMS, Merck, Astellas, Pfizer, Novartis with no personal financial compensation. LW participates in research with Merck, AZ, BMS, Roche, Pfizer with money going to her institution. NP reports personal fees from Ferring Pharmaceuticals, personal fees from AbbVie Pharmaceuticals, outside the submitted work. CA reports personal fees from Allergan, grants and personal fees from AstraZeneca, grants and personal fees from AbbVie, grants and personal fees from Paladin Labs, grants and personal fees from Sanofi, outside the submitted work. The remaining authors declare that the research was conducted in the absence of any commercial or financial relationships that could be construed as a potential conflict of interest.

## References

[1] Rosenthal SA Hu C Sartor O Gomella LG Amin MB Purdy J. Effect of chemotherapy with docetaxel with androgen suppression and radiotherapy for localized high-risk prostate cancer: the randomized phase III NRG oncology RTOG 0521 trial. J Clin Oncol. (2019) 37:1159–68. 10.1200/JCO.18.02158 30860948 PMC6506419

[2] Guttilla A Bortolus R Giannarini G Ghadjar P Zattoni F Gnech M. Multimodal treatment for high-risk prostate cancer with high-dose intensity-modulated radiation therapy preceded or not by radical prostatectomy, concurrent intensified-dose docetaxel and long-term androgen deprivation therapy: results of a prospective phase II trial. Radiat Oncol Lond Engl. (2014) 9:24. 10.1186/1748-717X-9-24 24423462 PMC3905924

[3] Hurwitz MD Harris J Sartor O Xiao Y Shayegan B Sperduto PW. Adjuvant radiation therapy, androgen deprivation, and docetaxel for high-risk prostate cancer postprostatectomy: results of NRG Oncology/RTOG study 0621. Cancer. (2017) 123:2489–96. 10.1002/cncr.30620 28323339 PMC5474197

[4] Jackson WC Feng FY Daignault S Hussain M Smith D Cooney K. A phase 2 trial of salvage radiation and concurrent weekly docetaxel after a rising prostate-specific antigen level after radical prostatectomy. Adv Radiat Oncol. (2016) 1:59–66. 10.1016/j.adro.2015.11.001 28799570 PMC5506748

[5] Rice SR Olexa G Hussain A Mannuel H Naslund MJ Amin P. A phase II study evaluating bone marrow-sparing, image-guided pelvic intensity-modulated radiotherapy (IMRT) with cesium-131 brachytherapy boost, adjuvant chemotherapy, and long-term hormonal ablation in patients with high risk, nonmetastatic prostate cancer. Am J Clin Oncol. (2019) 42:285–91. 10.1097/COC.0000000000000520 30676332 PMC7384590

[6] Zattoni F Morlacco A Matrone F Arcicasa M Buttazzi L Maruzzi D. Multimodal treatment for high risk locally advanced prostate cancer following radical prostatectomy and extended lymphadenectomy: results of a prospective cohort study with high-dose intensity-modulated radiation therapy, concurrent docetaxel and long-term androgen deprivation therapy. Minerva Urol Nefrol. (2019) 71:508–15. 10.23736/S0393-2249.19.03388-5 30957475

[7] Siegel RL Miller KD Jemal A Cancer Statistics, (2017). CA Cancer J Clin. (2017) 67:7–30. 10.3322/caac.21387 28055103

[8] Bandini M Marchioni M Pompe RS Tian Z Gandaglia G Fossati N. First North American validation and head-to-head comparison of four preoperative nomograms for prediction of lymph node invasion before radical prostatectomy. BJU Int. (2018) 121:592–9. 10.1111/bju.14074 29124911

[9] Pilepich MV Winter K Lawton CA Krisch RE Wolkov HB Movsas B. Androgen suppression adjuvant to definitive radiotherapy in prostate carcinoma-long-term results of phase III RTOG 85-31. Int J Radiat Oncol. (2005) 61:1285–90. 10.1016/j.ijrobp.2004.08.047 15817329

[10] Milas L Milas MM Mason KA. Combination of taxanes with radiation: preclinical studies. Semin Radiat Oncol. (1999) 9(2 Suppl 1):12–26. 10210536

[11] Hennequin C Giocanti N Favaudon V. Interaction of ionizing radiation with paclitaxel (Taxol) and docetaxel (Taxotere) in HeLa and SQ20B cells. Cancer Res. (1996) 56:1842–50. 8620502

[12] Mason KA Hunter NR Milas M Abbruzzese JL Milas L. Docetaxel enhances tumor radioresponse *in vivo*. Clin Cancer Res. (1997) 3(12 Pt 1):2431–8. 9815644

[13] Tannock IF de Wit R Berry WR Horti J Pluzanska A Chi KN Docetaxel plus prednisone or mitoxantrone plus prednisone for advanced prostate cancer. N Engl J Med. (2004) 351:1502–12. 10.1056/NEJMoa040720 15470213

[14] Kumar P Perrotti M Weiss R Todd M Goodin S Cummings K. Phase I trial of weekly docetaxel with concurrent three-dimensional conformal radiation therapy in the treatment of unfavorable localized adenocarcinoma of the prostate. J Clin Oncol. (2004) 22:1909–15. 10.1200/JCO.2004.02.001 15143084

[15] Wei JT Dunn RL Litwin MS Sandler HM Sanda MG. Development and validation of the expanded prostate cancer index composite (EPIC) for comprehensive assessment of health-related quality of life in men with prostate cancer. Urology. (2000) 56:899–905. 10.1016/S0090-4295(00)00858-X 11113727

[16] McLaughlin PW Liss AL Nguyen PL Assimos DG D'Amico AV Gottschalk AR. ACR appropriateness criteria® locally advanced, high-risk prostate cancer. Am J Clin Oncol. (2017) 40:1–10. 10.1097/COC.0000000000000354 28059930

[17] Kattan MW Wheeler TM Scardino PT. Postoperative nomogram for disease recurrence after radical prostatectomy for prostate cancer. J Clin Oncol Off J Am Soc Clin Oncol. (1999) 17:1499–507. 10.1200/JCO.1999.17.5.1499 10334537

[18] Sanfilippo NJ Taneja SS Chachoua A Lepor H Formenti SC. Phase I/II study of biweekly paclitaxel and radiation in androgen-ablated locally advanced prostate cancer. J Clin Oncol. (2008) 26:2973–8. 10.1200/JCO.2007.14.4105 18565883

